# Machine learning-based risk factor analysis of adverse birth outcomes in very low birth weight infants

**DOI:** 10.1038/s41598-022-16234-y

**Published:** 2022-10-01

**Authors:** Hannah Cho, Eun Hee Lee, Kwang-Sig Lee, Ju Sun Heo

**Affiliations:** 1 Department of Pediatrics, Korea University College of Medicine, Anam Hospital, 73 Goryeodae-ro, Seongbuk-gu, Seoul, 02841 Korea; 2 Department of Pediatrics, Korea University Anam Hospital, Seoul, Korea; 3 AI Center, Korea University College of Medicine, Anam Hospital, 73 Goryeodae-ro, Seongbuk-gu, Seoul, 02841 Korea

**Keywords:** Health care, Risk factors

## Abstract

This study aimed to analyze major predictors of adverse birth outcomes in very low birth weight (VLBW) infants including particulate matter concentration (PM_10_), using machine learning and the national prospective cohort. Data consisted of 10,423 VLBW infants from the Korean Neonatal Network database during January 2013–December 2017. Five adverse birth outcomes were considered as the dependent variables, i.e., gestational age less than 28 weeks, gestational age less than 26 weeks, birth weight less than 1000 g, birth weight less than 750 g and small-for-gestational age. Thirty-three predictors were included and the artificial neural network, the decision tree, the logistic regression, the Naïve Bayes, the random forest and the support vector machine were used for predicting the dependent variables. Among the six prediction models, the random forest had the best performance (accuracy 0.79, area under the receiver-operating-characteristic curve 0.72). According to the random forest variable importance, major predictors of adverse birth outcomes were maternal age (0.2131), birth-month (0.0767), PM_10_ month (0.0656), sex (0.0428), number of fetuses (0.0424), primipara (0.0395), maternal education (0.0352), pregnancy-induced hypertension (0.0347), chorioamnionitis (0.0336) and antenatal steroid (0.0318). In conclusion, adverse birth outcomes had strong associations with PM_10_ month as well as maternal and fetal factors.

## Introduction

Preterm birth brings a significant disease burden for infants and children in the world. On a yearly basis, fifteen million newborns are preterm on the globe and this is a major cause of one million deaths among those aged 0–4 years in the world^1,2^. The burden of preterm birth including its mortality and morbidity seems to be increasing in most countries^3^. Neonatal intensive care has advanced over the last few decades but extremely preterm (EP, less than 28 weeks of gestation) or extremely low birth weight (ELBW, less than 1000 g) infants have been subjected to death, neonatal complications or long-term neurodevelopmental impairment^4–6^, which presents a great challenge for the health care system as well^7^. Small for gestational age (SGA) is another significant contributor for the global burden of preterm birth^8,9^ and it is very important to identify the risk factors of these adverse birth outcomes above.

On the other hand, air pollution has become an important health issue in the world, increasing global mortality and morbidity. In 2016, ambient air pollution resulted in 4.2 million premature deaths worldwide^10,11^. This is particularly notable in a country such as Korea, where air pollution including particulate matter (PM) has registered rapid growth over the past few decades along with rapid industrialization and urbanization. Especially, maternal exposure to PM is reported to be a major cause of adverse health outcomes. Maternal exposure to PM_2.5_ (fine inhalable particles with diameters < 2.5 μm) or PM_10_ (inhalable particles with diameters < 10 μm) is found to have positive relationships with preterm birth^12–17^ and low birth weight^18–20^. However, little literature is available on associations between maternal exposure to PM and more adverse birth outcomes such as EP, ELBW or SGA. Furthermore, no endeavor has been made regarding the utilization of machine learning for the prediction of adverse birth outcomes among very low birth weight (VLBW, less than 1500 g) infants. In this context, this study employed machine learning and a national prospective cohort registry database to examine main predictors of adverse birth outcomes in VLBW infants including PM_10_ as a marker of air pollution.

## Results

Descriptive statistics are shown for participants’ adverse birth outcomes and their predictors in Table 1. Among 10,423 participants, 3961 (38.0%), 1919 (18.4%), 3960 (38.0%), 1658 (15.9%) and 2242 (21.5%) belonged to the categories of *GA* < 28, *GA* < 26, *BW* < 1000, *BW* < 750 and *SGA*, respectively. Indeed, the mean and standard deviation of maternal age were 31 and 4.28, respectively. Monthly PM_10_ (micrograms per cube meter) were 52 (January), 49 (February), 60 (March), 57 (April), 60 (May), 43 (June), 35 (July), 34 (August), 34 (September), 38 (October), 47 (November) and 48 (December). Yearly PM_10_ (micrograms per cube meter), temperature average (degree Celsius), temperature min (degree Celsius) and temperature max (degree Celsius) in 2013, 2014, 2015, 2016 and 2017 were: 48, 46, 48, 48 and 47 for yearly PM_10_; 12.85, 14.10, 13.85, 14.80 and 14.60 for yearly temperature average; 7.35, 8.55, 8.50, 10.05 and 9.30 for yearly temperature min; and 19.05, 20.50, 20.05, 20.45 and 20.65 for yearly temperature max. The results of univariate analysis were presented in Table 2. The *P* values were smaller than 0.10 for the following variables: birth-month with *GA* < 28, *GA* < 26, *BW* < 750 and *SGA*; sex (male) with *GA* < 28, *GA* < 26, *BW* < 1000 and *BW* < 750 (positive relationship); number of fetuses with *GA* < 28, *GA* < 26, *BW* < 1000 and *BW* < 750 (negative relationship); maternal age with *SGA* (positive relationship); and PM_10_ month with *BW* < 1000 and *BW* < 750 (positive relationship).

**Table 1:** Descriptive statistics: adverse birth outcomes and categorical predictors.

Variable	*n*	%
Gestational age < 28 weeks	3961	38.0
Gestational age < 26 weeks	1919	18.4
Birth weight < 1000 g	3960	38.0
Birth weight < 750 g	1658	15.9
Small-for-gestational-age	2242	21.5
Sex: Male	5270	50.6
**Birth-year**		
2013	1395	13.4
2014	2126	20.4
2015	2399	23.0
2016	2365	22.7
2017	2138	20.5
Birth-Season: Spring	2535	24.3
Birth-Season: Summer	2623	25.2
Birth-Season: Autumn	2759	26.5
Birth-Season: Winter	2506	24.0
**Number of fetuses**		
1	6761	64.9
2	3247	31.2
3	400	3.8
4 or more	15	0.1
In vitro fertilization	2403	23.1
Gestational DM	830	8.0
Overt DM	114	1.1
Pregnancy-induced hypertension	1986	19.2
Chronic hypertension	223	2.1
Chorioamnionitis	3019	29.0
PROM	3661	35.1
PROM > 18 h	2467	23.7
Antenatal steroid	8310	79.7
Cesarean section	8106	77.8
Oligohydramnios	1413	13.6
Polyhydramnios	153	1.5
Primipara	6497	62.3
**Maternal education**		
Elementary	29	0.3
Junior high	122	1.2
Senior high	1931	18.5
College or higher	8341	80.0
**Maternal citizenship**		
Korea	10,023	96.2
China	124	1.2
Vietnam	122	1.2
Philippines	62	0.6
Cambodia	35	0.3
Other	57	0.2
**Paternal education**		
Elementary	8	0.1
Junior high	52	0.5
Senior high	1280	12.3
College or higher	9083	87.1
**Paternal citizenship**		
Korea	10,191	97.8
Vietnam	79	0.8
Philippines	19	0.2
Cambodia	7	0.1
China	5	0.0
Other	122	1.2
Unmarried	216	2.1
Congenital infection	128	1.2

DM, diabetes mellitus; PROM, prelabor rupture of membrane.

**Table 2:** Univariate analysis.

Variable	GA < 28			GA < 26			BW < 1000			BW < 750			SGA		
	No (n = 6462)	Yes (n = 3961)	*P* value	No (n = 8504)	Yes (n = 1919)	*P* value	No (n = 6463)	Yes (n = 3960)	*P* value	No (n = 8765)	Yes (n = 1658)	*P* value	No (n = 8181)	Yes (n = 2242)	*P* value
Birth-Month (%)			0.065*			0.069*			0.115			0.002*			0.012*
January	7.6	7.7		7.7	7.7		7.4	8.1		7.5	8.4		7.3	8.9	
February	7.3	6.1		7.1	5.8		7.3	6.1		7.1	5.5		7.0	6.4	
March	8.3	8.4		8.3	8.7		8.1	8.7		8.0	9.9		7.9	10.0	
April	7.7	8.3		7.8	8.5		7.6	8.5		7.9	8.1		7.8	8.2	
May	7.7	8.6		7.8	9.6		7.8	8.5		7.8	9.6		8.2	7.8	
June	7.6	8.4		7.9	7.4		8.0	7.7		7.9	7.8		8.1	7.4	
July	8.0	9.0		8.3	8.7		8.4	8.4		8.4	8.3		8.5	8.1	
August	9.2	8.3		8.8	9.2		9.0	8.6		8.9	8.3		8.8	9.1	
September	8.4	7.9		8.3	7.9		8.1	8.3		8.3	7.8		8.2	8.2	
October	8.9	9.1		9.0	8.5		9.0	8.8		8.9	9.0		8.9	9.1	
November	9.4	9.3		9.2	10.0		9.3	9.3		9.2	9.8		9.7	8.2	
December	9.9	9.0		9.9	8.0		10.0	8.9		9.9	7.4		9.8	8.8	
Male (%)	49.0	53.0	< 0.001*	50.0	52.5	0.051*	51.8	48.6	0.001*	51.5	45.6	< 0.001*	50.5	50.7	0.892
N-Fetuses (%)			< 0.001*			0.023*			< 0.001*			0.078*			0.704
1	62.4	68.9		64.5	67.4		62.9	68.0		64.5	67.0		64.6	65.8	
2	32.8	28.4		31.4	29.6		32.4	29.1		31.4	29.6		31.4	30.4	
3	4.5	2.7		4.0	3.1		4.4	2.9		3.9	3.4		3.9	3.6	
4 or more	0.2	0.0		0.2	0.0		0.2	0.0		0.2	0.0		0.1	0.2	
Maternal age (mean ± SD)	32.99 ± 4.28	33.05 ± 4.27	0.453	33.02 ± 4.28	32.95 ± 4.29	0.548	32.97 ± 4.30	33.08 ± 4.24	0.173	32.99 ± 4.28	33.11 ± 4.25	0.315	32.96 ± 4.28	33.21 ± 4.28	0.014*
PM_10_ Month (mean ± SD)	46.12 ± 9.28	46.31 ± 9.34	0.317	46.14 ± 9.27	46.44 ± 9.50	0.201	46.07 ± 9.24	46.40 ± 9.42	0.083*	46.09 ± 9.26	46.73 ± 9.55	0.012*	46.12 ± 9.26	46.48 ± 9.48	0.110

BW, birth weight (grams); GA, gestational age (weeks); N-fetuses, number of fetuses, PM, particulate matter; SGA, small-for-gestational age.

*P < 0.10 chi-square test for the equality of proportions “Yes” or T test for the equality of means.

**Figure 1:**
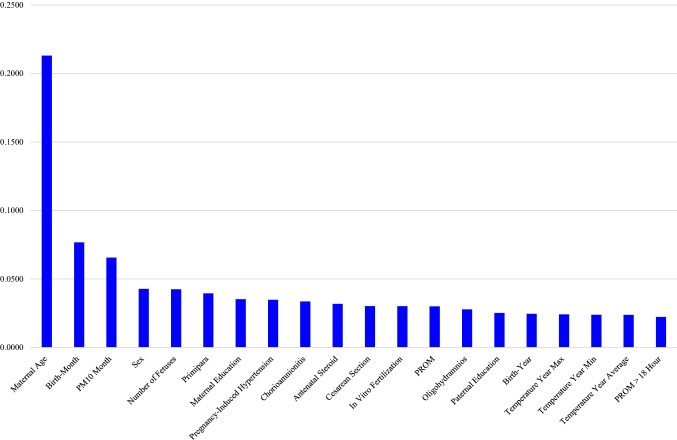
Random forest variable importance plots for adverse birth outcomes: PM_10_ included. PM, particulate matter; PROM, prelabor rupture of membranes.

The performance of the random forest was the best among the six models in this study (Table 3). Its areas under the receiver-operating-characteristic curve, 0.73 (PM_10_ excluded) and 0.72 (PM_10_ included), were higher than its logistic-regression counterparts, 0.69 (PM_10_ excluded) and 0.68 (PM_10_ included). Based on random forest variable importance in Table 4 (PM_10_ excluded), main predictors of adverse birth outcomes were maternal age (0.2276), birth-year (0.1216), birth-month (0.1165), sex (0.0415), number of fetuses (0.0404), primipara (0.0373), maternal education (0.0361), pregnancy-induced hypertension (0.0325), chorioamnionitis (0.0319) and antenatal steroid (0.0307). Likewise, according to random forest variable importance in Table 5 (PM_10_ included), major predictors of adverse birth outcomes were maternal age (0.2131), birth-month (0.0767), PM_10_ month (0.0656), sex (0.0428), number of fetuses (0.0424), primipara (0.0395), maternal education (0.0352), pregnancy-induced hypertension (0.0347), chorioamnionitis (0.0336) and antenatal steroid (0.0318) (Fig. 1). These values were the pooled outcome for the five adverse birth outcomes. It was found that the variable importance of birth-year disappears after the inclusion of PM_10_ month in the model. Finally, Table 6 helps to understand how variable importance rankings vary among different adverse birth outcomes. The ranking of a top-5 (or top-10) predictor was highlighted with the color of orange (or mild blue) in each column of the five adverse birth outcomes in the table. Maternal age, birth-month and PM_10_ month were the first, second and third most important predictors across board. However, some predictors were outside the top 5 on average but within the top 5 in certain adverse birth outcomes: primipara (6th on average) ranked 4th and 5th in *BW* < 1000 and *BW* < 750, respectively; pregnancy-induced hypertension (8th on average) ranked 4th in *SGA*; and chorioamnionitis (9th on average) ranked 4th in *GA* < 28. Also, it needs to be noted that maternal education ranked within the top 10 across board.

**Table 3:** Model Performance.

Model	GA < 28		GA < 26		BW < 1000		BW < 750		SGA		Average	
	Accuracy	AUC	Accuracy	AUC	Accuracy	AUC	Accuracy	AUC	Accuracy	AUC	Accuracy	AUC
**PM**_**10**_** excluded**												
LR	0.66	0.69	0.82	0.69	0.62	0.60	0.84	0.61	0.80	0.79	0.80	0.69
DT	0.62	0.61	0.75	0.61	0.58	0.56	0.75	0.56	0.74	0.62	0.74	0.61
NB	0.64	0.67	0.70	0.67	0.61	0.59	0.78	0.60	0.68	0.76	0.68	0.67
RF	0.68	0.73	0.83	0.74	0.63	0.63	0.84	0.64	0.79	0.77	0.79	0.73
SVM	0.62	0.64	0.82	0.56	0.62	0.52	0.84	0.52	0.79	0.67	0.79	0.56
ANN	0.62	0.50	0.82	0.50	0.62	0.50	0.84	0.50	0.79	0.50	0.79	0.50
**PM**_**10**_** included**												
LR	0.65	0.68	0.81	0.69	0.62	0.59	0.84	0.61	0.80	0.78	0.80	0.68
DT	0.63	0.61	0.75	0.61	0.59	0.57	0.75	0.56	0.73	0.61	0.73	0.61
NB	0.64	0.66	0.69	0.67	0.61	0.59	0.79	0.60	0.67	0.75	0.67	0.66
RF	0.68	0.72	0.82	0.73	0.63	0.63	0.84	0.64	0.79	0.76	0.79	0.72
SVM	0.62	0.64	0.81	0.53	0.62	0.51	0.84	0.50	0.79	0.60	0.79	0.53
ANN	0.62	0.54	0.82	0.49	0.62	0.51	0.84	0.48	0.78	0.51	0.78	0.51

ANN artificial neural network; AUC, area under the receiver-operating-characteristic curve; BW, birth weight (grams); DT, decision tree; GA, gestational age (weeks); LR, logistic regression; NB, naive bayes; PM, particulate matter; RF, random forest—1000 trees; SGA, small-for-gestational age; SVM, support vector machine.

**Table 4:**
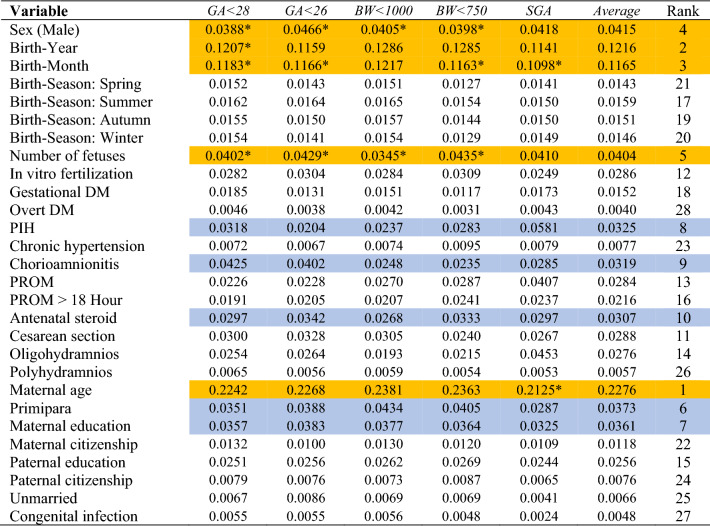
Random forest variable importance for adverse birth outcomes: PM_10_ excluded.

The ranking of a top-5 predictor was highlighted with the color of orange and 6–10 predictor was highlighted with the color of mild blue.

BW, birth weight (grams); DM, diabetes mellitus; GA, gestational age (weeks); PIH, pregnancy-induced hypertension; PM, particulate matter; PROM, prelabor rupture of membranes; SGA, small-for-gestational age.

*P < 0.10 chi-square or T Test (Table 2).

**Table 5:**
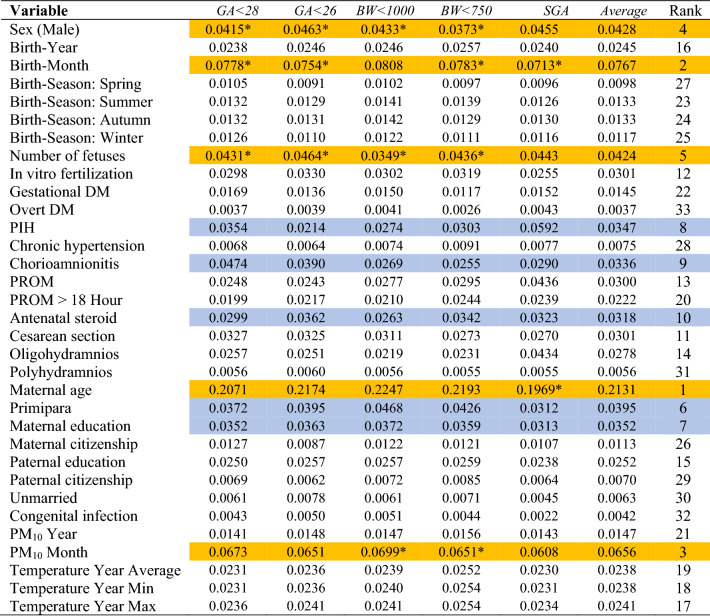
Random forest variable importance for adverse birth outcomes: PM_10_ included.

The ranking of a top-5 predictor was highlighted with the color of orange and 6–10 predictor was highlighted with the color of mild blue.

BW, birth weight (grams); DM, diabetes mellitus; GA, gestational age (weeks); PIH, pregnancy-induced hypertension; PM, particulate matter; PROM, prelabor rupture of membranes; SGA, small-for-gestational age.

*P < 0.10 chi-square or T test (Table 2).

**Table 6:**
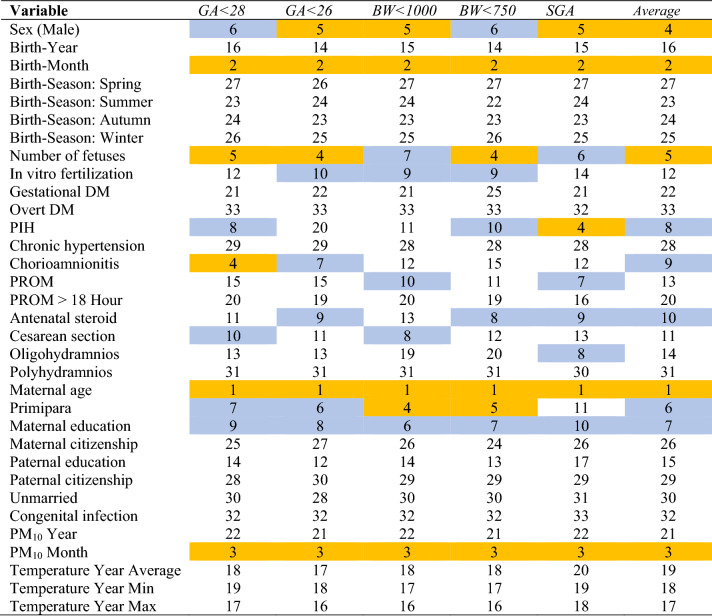
Random forest variable importance rankings for adverse birth outcomes: PM_10_ included.

The ranking of a top-5 predictor was highlighted with the color of orange and 6–10 predictor was highlighted with the color of mild blue.

BW, birth weight (grams); DM, diabetes mellitus; GA, gestational age (weeks); PIH, pregnancy-induced hypertension; PM, particulate matter; PROM, prelabor rupture of membranes; SGA, small-for-gestational age.

*P < 0.10 chi-square or T test (Table 2).

## Discussion

This study used machine learning to provide the most comprehensive investigation for the predictors of five adverse birth outcomes, using a national prospective cohort registry (KNN) database in VLBW infants. Among the six prediction models for adverse birth outcomes, the random forest had the best performance (accuracy 0.79, area under the receiver-operating-characteristic curve 0.72). According to the random forest variable importance, major predictors of adverse birth outcomes were maternal age, birth-month, PM_10_ month, sex, number of fetuses, primipara, maternal education, pregnancy-induced hypertension, chorioamnionitis and antenatal steroid.

In this study, birth month and PM_10_ had affect to EP, ELBW and SGA. Environmental changes according to the month such as air pollution, ambient temperature, the synthesis of vitamin D from sunlight could affect the adverse birth outcomes. Recently, existing literature reports the positive association of adverse birth outcomes with maternal exposure to PM during specific trimester or entire pregnancy^21,22^. A meta-analysis study reported that PM_10_ was found to increase extremely preterm birth^21^, and in particular, the incidence of preterm birth increased in Asia with high concentration of air pollution^22^. Unlike previous studies, this study found that high air pollution exposure in the birth month, rather than specific trimester or entire pregnancy period, was strongly associated with adverse birth outcomes. This result was consistent with some previous studies that maternal exposure to PM during the birth month was a major risk factor for adverse birth outcomes^23,24^. A time-series study conducted by Liu et al. showed that short term exposure to air pollution one week before childbirth increased preterm birth^23^. In addition, Trasande et al.'s air pollution research model showed that PM_2.5_ concentration in the birth month was associated with low birth weight and VLBW^24^. A possible pathway between maternal exposure to PM and adverse birth outcome would be oxidative stress, placental dysfunction, endothelial dysfunction and abnormal fetal growth^25,26^. But more examination is to be done on this issue.

Sex (male) was the fourth most important predictor for adverse birth outcomes in VLBW infants in this study. This result is consistent with a recent review^27^, which suggests the following mechanism: In the presence of a male fetus the trophoblast causes a more pro-inflammatory environment, and the maturation of the fetal hypothalamic–pituitary–adrenal axis and the expression of placental genes follow sex-dependent patterns.

The number of fetuses was found to have a negative association with adverse birth outcomes in VLBW infants in this study. This finding does not agree with a recent review, which reports that the number of fetuses is a strong risk factor for adverse birth outcome^28^. One way to explain this discrepancy is that more active prenatal screening and monitoring were done in the case of multiple fetuses in this study: Pregnancy with multiple fetuses often involves in vitro fertilization, which can associate with high socioeconomic status and active prenatal care^29^.

Maternal age was the first most important predictor for adverse birth outcomes in VLBW infants but the result of univariate analysis was significant only for SGA in this study. The participants of this study were limited to VLBW infants and more investigation based on broader participants is to be done for more conclusive result.

Some predictors were outside the top 5 on average but within the top 5 in certain adverse birth outcomes in this study: pregnancy-induced hypertension (eighth on average) ranked fourth in *SGA*; and chorioamnionitis (ninth on average) ranked fourth in *GA* < 28. Chorioamnionitis is considered to be a major predictor of preterm birth^30,31^. It is expected to encourage pro-inflammatory cytokines, uterine contraction and preterm labor; or it is supposed to promote matrix metalloproteinase activation, fetal membrane degradation and preterm birth^32^. Also, early onset preeclampsia is reported to cause abnormal placentation and spiral artery remodeling, which in turn lead to restriction on blood flow to uterine arteries and abnormal fetal growth^33–35^.

### Limitations

Firstly, this study did not analyze possible mediating effects among predictors. Secondly, this study adopted the binary categories of adverse birth outcomes (no, yes), which can be extended to multiple categories with more clinical insights. Thirdly, it was beyond the scope of this study to investigate a variety of mechanisms between PM and adverse birth outcomes. Little effort has been made and more examination is needed in this direction. Fourthly, synthesizing different modes of machine learning methods for different types of adverse birth outcome data would break new ground on this topic. Fifthly, this study did not consider indoor enviromental factors, which were reported as major predictors of adverse birth outcomes along with PM^20,36^.

### Conclusions

The current study is the first to evaluate the predictors including PM_10_ month on adverse birth outcomes in VLBW infants using machine learning. Adverse birth outcomes such as EP, ELBW or SGA have strong associations with PM_10_ month as well as maternal and fetal predictors among the VLBW infants. For the prevention of adverse effects on birth outcomes, clinical and/or policy measures are needed regarding these predictors.

## Methods

### Participants and variables

Data consisted of 10,423 VLBW infants from the Korean Neonatal Network (KNN) database during January 2013-December 2017. The KNN started on April 2013 as a national prospective cohort registry of VLBW infants admitted or transferred to neonatal intensive care units across South Korea (It covers 74 neonatal intensive care units now). It collects the perinatal and neonatal data of VLBW infants based on a standardized operating procedure^37^.

Five adverse birth outcomes were considered as binary dependent variables (no, yes), i.e., gestational age less than 28 weeks (*GA* < 28), GA less than 26 weeks (*GA* < 26), birth weight less than 1000 g (*BW* < 1000), BW less than 750 g (*BW* < 750) and SGA. Thirty-three predictors were included: sex—male (no, yes), birth-year (2013, 2014, 2015, 2016, 2017), birth-month (1, 2, …, 12), birth-season-spring (no, yes), birth-season-summer (no, yes), birth-season-autumn (no, yes), birth-season-winter (no, yes), number of fetuses (1, 2, 3, 4 or more), in vitro fertilization (no, yes), gestational diabetes mellitus (no, yes), overt diabetes mellitus (no, yes), pregnancy-induced hypertension (no, yes), chronic hypertension (no, yes), chorioamnionitis (no, yes), prelabor rupture of membranes (no, yes), prelabor rupture of membranes > 18 h (no, yes), antenatal steroid (no, yes), cesarean section (no, yes), oligohydramnios (no, yes), polyhydramnios (no, yes), maternal age (years), primipara (no, yes), maternal education (elementary, junior high, senior high, college or higher), maternal citizenship (Korea, Vietnam, China, Philippines, Japan, Cambodia, United States, Thailand, Mongolia, Other), paternal education (elementary, junior high, senior high, college or higher), paternal citizenship (Korea, Vietnam, China, Philippines, Japan, Cambodia, United States, Thailand, Mongolia, Other), unmarried (no, yes), congenital infection (no, yes), PM_10_ year (PM_10_ for each year), PM_10_ month (PM_10_ for each birth-month), temperature average (for each year), temperature min (for each year) and temperature max (for each year). PM_10_ and temperature data came from the Korea Meteorological Administration (PM_10_ https://data.kma.go.kr/data/climate/selectDustRltmList.do?pgmNo=68; temperature https://web.kma.go.kr/weather/climate/past_cal.jsp). The definition of each variable is given in Text S1, supplementary text.

### Statistical analysis

The artificial neural network, the decision tree, the logistic regression, the Naïve Bayes, the random forest and the support vector machine were used for predicting preterm birth^38–43^. A decision tree includes three elements, i.e., a test on an independent variable (intermediate note), an outcome of the test (branch) and a value of the dependent variable (terminal node). A naïve Bayesian classifier performs classification on the basis of Bayes’ theorem. Here, the theorem states that the probability of the dependent variable given certain values of independent variables can be calculated based on the probabilities of the independent variables given a certain value of the dependent variable. A random forest is a collection of many decision trees, which make majority votes on the dependent variable (“bootstrap aggregation”). Let us take a random forest with 1000 decision trees as an example. Let us assume that original data includes 10,000 participants. Then, the training and test of this random forest takes two steps. Firstly, new data with 10,000 participants is created based on random sampling with replacement, and a decision tree is created based on this new data. Here, some participants in the original data would be excluded from the new data and these leftovers are called out-of-bag data. This process is repeated 1000 times, i.e., 1000 new data are created, 1000 decision trees are created and 1000 out-of-bag data are created. Secondly, the 1000 decision trees make predictions on the dependent variable of every participant in the out-of-bag data, their majority vote is taken as their final prediction on this participant, and the out-of-bag error is calculated as the proportion of wrong votes on all participants in the out-of-bag data^38,39^.

A support vector machine estimates a group of “support vectors”, that is, a line or space called “hyperplane”. The hyperplane separates data with the greatest gap between various sub-groups. An artificial neural network consists of “neurons”, information units combined through weights. In general, the artificial neural network includes one input layer, one, two or three intermediate layers and one output layer. Neurons in a previous layer link with “weights” in the next layer (Here, these weights denote the strengths of linkages between neurons in a previous layer and their next-layer counterparts). This “feedforward” operation begins from the input layer, runs through intermediate layers and ends in the output layer. Then, this process is followed by learning: These weights are updated according to their contributions for a gap between the actual and predicted final outputs. This “backpropagation” operation begins from the output layer, runs through intermediate layers and ends in the input layer. The two processes are repeated until the performance measure reaches a certain limit^38,39^. Data on 10,423 observations with full information were divided into training and validation sets with a 70:30 ratio (7296 vs. 3127). Accuracy, a ratio of correct predictions among 3127 observations, was employed as a standard for validating the models. Random forest variable importance, the contribution of a certain variable for the performance (GINI) of the random forest, was used for examining major predictors of adverse birth outcomes in VLBW infants including PM_10_. The random split and analysis were repeated 50 times then its average was taken for external validation^44,45^. R-Studio 1.3.959 (R-Studio Inc.: Boston, United States) was employed for the analysis during August 1, 2021–September 30, 2021.

### Ethic statement

The KNN registry was approved by the institutional review board (IRB) at each participating hospital (IRB No. of Korea University Anam Hospital: 2013AN0115). Informed consent was obtained from the parent(s) of each infant registered in the KNN. All methods were carried out in accordance with the IRB-approved protocol and in compliance with relevant guidelines and regulations.

The names of the institutional review board of the KNN participating hospitals were as follows: The institutional review board of Gachon University Gil Medical Center, The Catholic University of Korea Bucheon ST. Mary’s Hospital, The Catholic University of Korea Seoul ST. Mary’s Hospital, The Catholic University of Korea ST. Vincent’s Hospital, The Catholic University of Korea Yeouido ST. Mary’s Hospital, The Catholic University of Korea Uijeongbu ST. Mary’s Hospital, Gangnam Severance Hospital, Kyung Hee University Hospital at Gangdong, GangNeung Asan Hospital, Kangbuk Samsung Hospital, Kangwon National University Hospital, Konkuk University Medical Center, Konyang University Hospital, Kyungpook National University Hospital, Gyeongsang National University Hospital, Kyung Hee University Medical center, Keimyung University Dongsan Medical Center, Korea University Guro Hospital, Korea University Ansan Hospital, Korea University Anam Hospital, Kosin University Gospel Hospital, National Health Insurance Service Iilsan Hospital, Daegu Catholic University Medical Center, Dongguk University Ilsan Hospital, Dong-A University Hospital, Seoul Metropolitan Government-Seoul National University Boramae Medical Center, Pusan National University Hospital, Busan ST. Mary’s Hospital, Seoul National University Bundang Hospital, Samsung Medical Center, Samsung Changwon Medical Center, Seoul National University Hospital, Asan Medical Center, Sungae Hospital, Severance Hospital, Soonchunhyang University Hospital Bucheon, Soonchunhyang University Hospital Seoul, Soonchunhyang University Hospital Cheonan, Ajou University Hospital, Pusan National University Children’s Hospital, Yeungnam University Hospital, Ulsan University Hospital, Wonkwang University School of Medicine & Hospital, Wonju Severance Christian Hospital, Eulji University Hospital, Eulji General Hospital, Ewha Womans University Medical.

Center, Inje University Busan Paik Hospital, Inje University Sanggye Paik Hospital, Inje University Ilsan Paik Hospital, Inje University Haeundae Paik Hospital, Inha University Hospital, Chonnam National University Hospital, Chonbuk National University Hospital, Cheil General Hospital & Women’s Healthcare Center, Jeju National University Hospital, Chosun University Hospital, Chung-Ang University Hospital, CHA Gangnam Medical Center, CHA University, CHA Bundang Medical Center, CHA University, Chungnam National University Hospital, Chungbuk National University, Kyungpook National University Chilgok Hospital, Kangnam Sacred Heart Hospital, Kangdong Sacred Heart Hospital, Hanyang University Guri Hospital, and Hanyang University Medical Center.

## Supplementary Information

Supplementary Information.

## Data Availability

The code and data used for this study are available from the corresponding author upon reasonable request and under the permission of the Korean Neonatal Network and the Korea Centers for Disease Control and Prevention.

## References

[1] Liu L (2016). Global, regional, and national causes of under-5 mortality in 2000–15: An updated systematic analysis with implications for the sustainable development goals. Lancet.

[2] World Health Organization. News: Preterm Birth. http://www.who.int/news-room/fact-sheets/detail/preterm-birth (2018).

[3] Harrison MS, Goldenberg RL (2016). Global burden of prematurity. Semin. Fetal Neonatal Med..

[4] Bell EF (2022). Mortality, in-hospital morbidity, care practices, and 2-year outcomes for extremely preterm infants in the US, 2013–2018. JAMA.

[5] Johnson S, Marlow N (2017). Early and long-term outcome of infants born extremely preterm. Arch. Dis. Child..

[6] Glass HC (2015). Outcomes for extremely premature infants. Anesth. Analg..

[7] Holsti A, Adamsson M, Hägglöf B, Farooqi A, Serenius F (2017). Chronic conditions and health care needs of adolescents born at 23 to 25 weeks' gestation. Pediatrics.

[8] Jensen EA (2019). Adverse effects of small for gestational age differ by gestational week among very preterm infants. Arch. Dis. Child. Fetal Neonatal Ed..

[9] Boghossian NS, Geraci M, Edwards EM, Horbar JD (2018). Morbidity and mortality in small for gestational age infants at 22 to 29 weeks' gestation. Pediatrics.

[10] World Health Organization, 2016. Ambient (outdoor) air quality and health. Fact sheet. Retrieved from http://www.who.int/mediacentre/factsheets/fs313/en/ (2016).

[11] Lim SS (2012). A comparative risk assessment of burden of disease and injury attributable to 67 risk factors and risk factor clusters in 21 regions, 1990–2010: A systematic analysis for the Global Burden of Disease Study 2010. Lancet.

[12] Padula AM (2014). Traffic-related air pollution and risk of preterm birth in the San Joaquin Valley of California. Ann. Epidemiol..

[13] DeFranco E (2016). Exposure to airborne particulate matter during pregnancy is associated with preterm birth: A population-based cohort study. Environ. Health.

[14] Mendola P (2019). Air pollution and preterm birth: Do air pollution changes over time influence risk in consecutive pregnancies among low-risk women?. Int. J. Environ. Res. Public Health.

[15] Lavigne E (2016). Ambient air pollution and adverse birth outcomes: Differences by maternal comorbidities. Environ. Res..

[16] Qian Z (2016). Ambient air pollution and preterm birth: A prospective birth cohort study in Wuhan, China. Int. J Hyg. Environ. Health.

[17] Kim YJ (2019). Maternal exposure to particulate matter during pregnancy and adverse birth outcomes in the Republic of Korea. Int. J. Environ. Res. Public Health.

[18] Bell ML (2010). Prenatal exposure to fine particulate matter and birth weight: Variations by particulate constituents and sources. Epidemiology.

[19] Fleischer NL (2014). Outdoor air pollution, preterm birth, and low birth weight: Analysis of the world health organization global survey on maternal and perinatal health. Environ. Health Perspect..

[20] Lu C (2020). Combined effects of ambient air pollution and home environmental factors on low birth weight. Chemosphere.

[21] Ju L (2021). Maternal air pollution exposure increases the risk of preterm birth: Evidence from the meta-analysis of cohort studies. Environ. Res..

[22] Ye L (2018). Associations between maternal exposure to air pollution and birth outcomes: A retrospective cohort study in Taizhou, China. Environ. Sci. Pollut. Res. Int..

[23] Liu WY (2018). Association between ambient air pollutants and preterm birth in Ningbo, China: A time-series study. BMC Pediatr..

[24] Trasande L, Wong K, Roy A, Savitz DA, Thurston G (2013). Exploring prenatal outdoor air pollution, birth outcomes and neonatal health care utilization in a nationally representative sample. J. Expo. Sci. Environ. Epidemiol..

[25] Glinianaia SV, Rankin J, Bell R, Pless-Mulloli T, Howel D (2004). Particulate air pollution and fetal health: A systematic review of the epidemiologic evidence. Epidemiology.

[26] Backes CH, Nelin T, Gorr MW, Wold LE (2013). Early life exposure to air pollution: How bad is it?. Toxicol. Lett..

[27] Challis J, Newnham J, Petraglia F, Yeganegi M, Bocking A (2013). Fetal sex and preterm birth. Placenta.

[28] Dodd JM, Grivell RM, OBrien CM, Dowswell T, Deussen AR (2017). Prenatal administration of progestogens for preventing spontaneous preterm birth in women with a multiple pregnancy. Cochrane Database Syst. Rev..

[29] Heo JS, Lee HJ, Lee MH, Choi CW (2019). Comparison of neonatal outcomes of very low birth weight infants by mode of conception: In vitro fertilization versus natural pregnancy. Fertil. Steril..

[30] Goldenberg RL, Culhane JF, Iams JD, Romero R (2008). Epidemiology and causes of preterm birth. Lancet.

[31] DiGiulio DB (2008). Microbial prevalence, diversity and abundance in amniotic fluid during preterm labor: A molecular and culture-based investigation. PLoS ONE.

[32] Jain VG, Willis KA, Jobe A, Ambalavanan N (2022). Chorioamnionitis and neonatal outcomes. Pediatr. Res..

[33] Sugimoto H (2003). Neutralization of circulating vascular endothelial growth factor (VEGF) by anti-VEGF antibodies and soluble VEGF receptor 1 (sFlt-1) induces proteinuria. J. Biol. Chem..

[34] Eremina V (2003). Glomerular-specific alterations of VEGF-A expression lead to distinct congenital and acquired renal diseases. J. Clin. Invest..

[35] Eremina V (2008). VEGF inhibition and renal thrombotic microangiopathy. N. Engl. J. Med..

[36] Lu C (2021). Effect of outdoor air pollution and indoor environmental factors on small for gestational age. Build. Environ..

[37] Chang YS, Ahn SY, Park WS (2013). The establishment of the Korean Neonatal Network (KNN). Neonatal Med..

[38] Lee KS, Ahn KH (2019). Artificial neural network analysis of spontaneous preterm labor and birth and its major determinants. J. Korean Med. Sci..

[39] Lee KS, Ahn KH (2020). Application of artificial intelligence in early diagnosis of spontaneous preterm labor and birth. Diagnostics (Basel).

[40] Park EK (2019). Machine learning approaches to radiogenomics of breast cancer using low-dose perfusion computed tomography: Predicting prognostic biomarkers and molecular subtypes. Sci. Rep..

[41] Lee JY (2021). Radiomic machine learning for predicting prognostic biomarkers and molecular subtypes of breast cancer using tumor heterogeneity and angiogenesis properties on MRI. Eur. Radiol..

[42] Lee KS, Song IS, Kim ES, Ahn KH (2020). Determinants of spontaneous preterm labor and birth including gastroesophageal reflux disease and periodontitis. J. Korean Med. Sci..

[43] Lee KS (2021). Association of preterm birth with depression and particulate matter: Machine learning analysis using national health insurance data. Diagnostics (Basel).

[44] Ahn KH (2021). Predictors of newborn's weight for height: A machine learning study using nationwide multicenter ultrasound data. Diagnostics (Basel).

[45] Lee KS, Kim ES, Kim DY, Song IS, Ahn KH (2021). Association of gastroesophageal reflux disease with preterm birth: Machine learning analysis. J. Korean Med. Sci..

