# Supplementary material for: Machine learning-based risk factor analysis of adverse birth outcomes in very low birth weight infants
Source: Sci Rep. 2022 Oct 1;12:12119. doi: 10.1038/s41598-022-16234-y (PMC9526718; doi:10.1038/s41598-022-16234-y)
Supplement: Supplementary file 1 — Supplementary Information. [file 41598_2022_16234_MOESM1_ESM.pdf]

Air pollution and adverse birth outcomes in very low birth weight infants: machine learning analysis using a nationwide cohort data

Running title: Air pollution and adverse birth outcomes in very low birth weight infants

Hannah Cho,[1,2] Eun Hee Lee,[1] Kwang-Sig Lee [3*] and Ju Sun Heo [1,2*]

[1]Department of Pediatrics, Korea University College of Medicine, Seoul, Korea

[2]Department of Pediatrics, Korea University Anam Hospital, Seoul, Korea

[3]AI Center, Korea University College of Medicine, Anam Hospital, Seoul, Korea

* Address for Correspondence:

Kwang-Sig Lee, PhD

AI Center, Korea University College of Medicine, Anam Hospital

73 Goryeodae-ro, Seongbuk-gu, Seoul 02841, Korea

E-mail: ecophy@hanmail.net

Ju Sun Heo, MD, PhD

Department of Pediatrics, Korea University College of Medicine, Anam Hospital

73 Goryeodae-ro, Seongbuk-gu, Seoul 02841, Korea

E-mail: heojs08@korea.ac.kr

Supplemental Text 1. Variable Definitions

Small for gestational age was defined as birth weight below the 10th percentile according to the Fenton growth chart [1]. Gestational diabetes mellitus was defined as any degree of glucose intolerance with onset or first recognition during pregnancy. Pregnancy-induced hypertension referred to hypertension with onset in the latter part of pregnancy (> 20 weeks' gestation) followed by normalization of the blood pressure postpartum. Chorioamnionitis was defined as histologic chorioamnionitis [2]. Oligohydramnios (or polyhydramnios) was defined as amniotic fluid index < 5cm (or > 24cm).

References

1 Fenton, T. R. & Kim, J. H. A systematic review and meta-analysis to revise the Fenton growth chart for preterm infants. *BMC Pediatr* **13**, 59, doi:10.1186/1471-2431-13-59 (2013).

2 Yoon, B. H. *et al.* Amniotic fluid interleukin-6: a sensitive test for antenatal diagnosis of acute inflammatory lesions of preterm placenta and prediction of perinatal morbidity. *Am J Obstet Gynecol* **172**, 960-970, doi:10.1016/0002-9378(95)90028-4 (1995).
